# Fluorescence indocyanine green (ICG) for sentinel-lymph-node mapping in colorectal cancer: a systematic review

**DOI:** 10.1007/s00423-025-03786-6

**Published:** 2025-07-01

**Authors:** Alexis Litchinko, Jeremy Meyer, Leo Buhler, Frederic Ris, Michel Adamina

**Affiliations:** 1 Division of Digestive Surgery, University Hospitals of Geneva, Rue Gabrielle-Perret-Gentil 4, 1211 Genève 14, Switzerland; 2 Faculty of Science, HFR Fribourg – Cantonal Hospital, Fribourg, CH-1711 Switzerland; 3 Division of Digestive Surgery, University Hospitals of Fribourg, Chem. des Pensionnats 2/6, Fribourg, 1752 Switzerland

**Keywords:** Systematic review, Colorectal cancer, Indocyanine green, Sentinel lymph node mapping

## Abstract

**Objective:**

Modern surgical guidance in laparoscopic colon cancer procedures could be enhanced by visualizing lymphatic flow during surgery, already helping surgeons in determining the precise extent of digestive resection and could be useful in lymphadenectomy. Related to oncological procedure, lymphadenectomy is mandatory to assess the extension of the disease. To explore this approach, the objective of this review is to examine the use of indocyanine green fluorescence imaging for real-time in vivo identification of lymphatic flow and especially sentinel nodes in patients undergoing elective surgery for colorectal cancer.

**Methods:**

A systematic review was conducted to identify relevant studies on sentinel node mapping using indocyanine green (ICG) in colorectal cancer surgery. A comprehensive search was performed in electronic databases including PubMed, Embase, and Cochrane Library from inception to December 2024. The search strategy incorporated relevant keywords and MeSH terms, combining variations of “colorectal neoplasms,” “sentinel lymph node,” “indocyanine green,” and related terms. The search was limited to articles published in English language.

**Results:**

A total of 405 studies were identified across all databases. After screening, 45 full-text articles were assessed for eligibility, and 12 studies were ultimately included in the systematic review. ICG-FI has not yet demonstrated superiority over the standard blue dye technique. Moreover, a notable heterogeneity exists among the reported studies concerning ICG dosage, injection methods and the definition of positive LN status for sensitivity calculations, making direct comparisons challenging.

**Conclusion:**

Despite the potential shown with other surgical oncological resections, ICG-FI requires further investigation and standardization in protocols and indications to fully harness its capabilities for SLN detection in CRC, especially metastatic nodes. Larger patient populations should be considered in future research to comprehensively assess its efficacy. This systematic review highlights the heterogeneity and limitations of current evidence regarding ICG-FI for SLN detection in colorectal cancer. While preliminary results are encouraging, further well-designed prospective trials are required before routine clinical implementation can be recommended.

## Introduction

Colorectal cancer is a significant global health burden, representing the third most diagnosed cancer and the second leading cause of cancer-related deaths worldwide [1]. Adequate lymph node assessment is crucial for accurate staging and treatment planning in colorectal cancer patients [2, 3]. Traditionally, extensive lymph node dissection involving the removal of the entire mesocolon has been the standard approach [4]. However, recent advancements have focused on identifying sentinel lymph nodes (SLNs) to guide more targeted and tailored surgical interventions [5–7].

Sentinel node mapping, originally introduced in breast cancer, has gained attention as a promising technique in colorectal cancer management [8–10]. This technique involves the identification and possible biopsy of the first lymph nodes to receive lymphatic drainage from the tumor, thus providing valuable information about the lymph node status without the need for extensive dissection. Accurate identification of SLNs allows for more precise staging and may help reduce the risk of unnecessary lymphadenectomy and associated morbidity and mortality, especially in a previously identified fragile population [8, 11–13]. 

Indocyanine green (ICG), a fluorescent dye with high lymphatic affinity, has emerged as a valuable tool for sentinel node mapping in various cancer types and various surgical specialty [14–16]. In colorectal cancer, ICG has demonstrated potential as a safe and effective agent for visualizing lymphatic flow and identifying SLNs during surgery. Its near-infrared fluorescence properties enable real-time intraoperative imaging, enhancing the surgeon’s ability to identify and selectively remove the relevant lymph nodes [15, 17, 18]. The use of ICG offers several advantages, including its minimal invasiveness, rapid clearance from the body, and ability to visualize deep-seated nodes.

Several studies have investigated the utility of ICG for sentinel node mapping in colorectal cancer, but the overall evidence and clinical implications remain to be fully and clearly elucidated [19, 20]. Therefore, a systematic review to synthesize the available literature and evaluate the accuracy, sensitivity, specificity, and clinical outcomes associated with ICG-based sentinel node mapping in colorectal cancer is warranted. This review aims to provide a comprehensive analysis of the current evidence, exploring the technical aspects of ICG administration, the detection methods employed, and the impact of this approach on surgical decision-making and patient outcomes.

By systematically reviewing the existing studies, we aim to assess the feasibility, reliability, and diagnostic performance of ICG-based sentinel node mapping in colorectal cancer. Furthermore, we will explore the potential benefits and limitations of this technique, its impact on lymph node staging, and its implications for treatment strategies, including the potential for a more tailored surgical intervention [16].

Ultimately, a comprehensive evaluation of sentinel node mapping using ICG in colorectal cancer will provide valuable insights into the role of this technique in clinical practice. The findings of this systematic review may contribute to enhancing surgical precision, optimizing treatment algorithms, and improving patient outcomes in colorectal cancer management.

## Materials and methods

### Literature search strategy and study selection

A comprehensive search was performed in four electronic databases: PubMed/MEDLINE, Embase, Scopus, and the Cochrane Library, from inception to 31 December 2024. The search strategy combined relevant keywords and MeSH terms for “colorectal cancer,” “sentinel lymph node,” and “indocyanine green.” All searches were limited to English-language publications. This approach was conducted in accordance with PRISMA 2020 guidelines and followed the protocol registered in PROSPERO (CRD42023396720). The complete search strategy is provided in Table 1.

**Table 1:** Search strategy

Database	Search build	Occurrences
MEDLINE	(“Sentinel Node“[MeSH] OR “Sentinel“[Title/Abstract] OR “Sentinel Nodes“[Title/Abstract] OR “Lymph Nodes“[Title/Abstract]) AND (“Indocyanine Green“[MeSH] OR “ICG“[Title/Abstract]) AND (“Colorectal“[MeSH] OR “Colorectal Cancer“[Title/Abstract] OR “Colon Cancer“[Title/Abstract] OR “Rectal Cancer“[Title/Abstract])	13 (89)
EMBASE	(‘sentinel lymph node’/exp OR sentinel OR ‘lymph nodes’:ti, ab) AND (‘indocyanine green’/exp OR ICG: ti, ab) AND (‘colorectal cancer’/exp OR ‘colon cancer’:ti, ab OR ‘rectal cancer’:ti, ab)	0 (210)
COCHRANE CENTRAL	(Sentinel Node OR Sentinel Nodes OR Lymph Nodes) AND (Indocyanine Green OR ICG) AND (Colorectal Cancer OR Colon Cancer OR Rectal Cancer)	0 (19)
SCOPUS	(TITLE-ABS-KEY(“sentinel lymph node” OR “sentinel node” OR “lymph node”) AND (“indocyanine green” OR “ICG”) AND (“colorectal cancer” OR “colon cancer” OR “rectal cancer”))	0 (87)
Other sources		0

### Eligibility criteria & PICO framework

The eligibility of studies was defined according to the PICOS framework to ensure a standardized and reproducible selection process:

- Population (P): Adult patients undergoing elective surgery for histologically confirmed colorectal cancer.
- Intervention (I): Intraoperative administration of indocyanine green (ICG) for sentinel lymph node (SLN) mapping, with fluorescence imaging techniques used to visualize lymphatic drainage.
- Comparator (C): No comparator was required for study inclusion. Studies with or without a control group were considered eligible.
- Outcomes (O): Primary outcomes included SLN identification rate, sensitivity, specificity, and detection of aberrant drainage pathways. Secondary outcomes included procedural feasibility, safety, and potential oncologic implications.
- Study Design (S): Original clinical studies, including prospective or retrospective cohorts, feasibility trials, and case series involving ≥ 3 patients. Review articles, conference abstracts, case reports with fewer than three patients, animal studies, and non-English publications were excluded.

These predefined criteria were consistently applied during the selection process to ensure methodological rigor and transparency. Very small case series with fewer than three patients were excluded to reduce the risk of anecdotal reporting and to ensure a minimal threshold for procedural standardization. This exclusion criterion was chosen pragmatically to limit bias while still allowing the inclusion of small but methodologically transparent feasibility studies.

### Inclusion and exclusion criteria

Studies were included if they met the following criteria: (1) focused on sentinel node mapping using ICG in colorectal cancer, (2) included human participants with histologically confirmed colorectal cancer, (3) provided information on the technical aspects of ICG administration and detection, (4) reported outcomes related to the accuracy, sensitivity, specificity, or clinical implications of sentinel node mapping using ICG. Exclusion criteria encompassed: (1) non-original studies (e.g., reviews, case reports, editorials), (2) studies involving non-human subjects, (3) studies not published in English, (4) studies lacking sufficient data or information on the topic of interest. Exclusion criteria encompassed studies published in languages other than English, as well as those lacking the characteristics indicative of original research.

### Outcomes

This study primarily assessed the effectiveness of the SLN technique, encompassing its detection ability, sensitivity, and the incidence of false negative results. An effective SLN technique is identified when a sentinel lymph node is located using the fluorescent marker. The detection metric is calculated by dividing the instances of sentinel lymph node identification by the total number of conducted procedures. Precision is gauged by comparing the accurate predictions of lymph node status in the SLN mapping against the total instances where a sentinel lymph node was identified. Sensitivity refers to the proportion of procedures where a cancer-positive sentinel lymph node was found out of all the procedures that had a positive lymph node. Instances where a sentinel lymph node was incorrectly identified as cancer-free, yet another lymph node was cancerous, were marked as false negatives. Additionally, the study also examined the safety and practicality of the SLN technique, with safety being measured by the occurrence of adverse events and practicality determined by the noted operational complications. A meta-analysis of the aggregated results was not feasible due to the limited number of studies available and the heterogeneity of the outcomes.

### Data extraction

Two independent reviewers (A.L. and F.R.) performed the title and abstract screening of all records retrieved from the literature search, using the predefined eligibility criteria. Full-text articles were retrieved for studies that met the inclusion criteria or required further clarification. The same reviewers independently extracted data using a standardized data collection form. In case of any discrepancies during study selection or data extraction, a consensus was reached through discussion. If disagreement persisted, a third author (M.A.) was consulted to resolve the issue. This three-step process was conducted in accordance with the PRISMA 2020 guidelines.

### Risk of bias assessment

The risk of bias for each included non-randomized study was assessed using the ROBINS-I (Risk Of Bias In Non-randomised Studies – of Interventions) tool, which evaluates bias across seven domains: confounding, participant selection, classification of interventions, deviations from intended interventions, missing data, measurement of outcomes, and selection of reported results. Two authors (A.L. and F.R.) independently performed the assessment. Disagreements were resolved by consensus, and a third reviewer (Q.D.) acted as arbiter if necessary. Each domain was rated as having low, moderate, serious, or critical risk of bias, and an overall risk of bias was determined for each study. A summary table of the ROBINS-I assessment is presented in Table 2. Given the observational nature and methodological heterogeneity of the included studies, no study achieved a low risk of bias across all domains. Most studies were rated as moderate to serious overall risk.

**Table 2:**
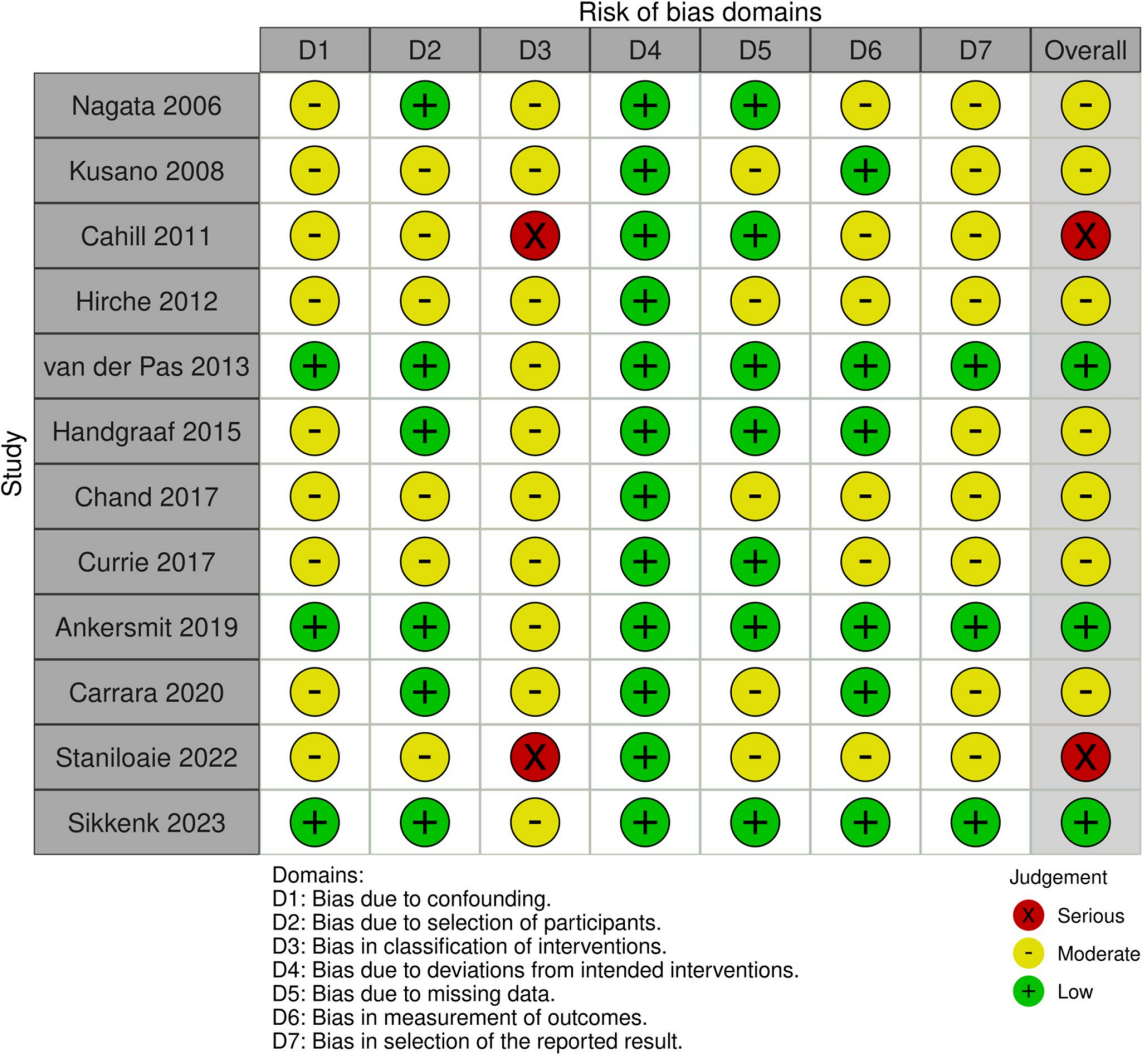
ROBINS-I risk of bias assessment across the 12 included studies

The GRADE framework was not formally applied due to the small number of studies, their exploratory designs, and the absence of pooled statistical data. However, the limitations in evidence quality are explicitly addressed in the Discussion.

### Data synthesis and analysis

Due to the heterogeneity of the included studies in terms of design, patient characteristics, surgical technique, injection site, and reporting methods, no meta-analysis was performed. A qualitative synthesis was conducted. Data extracted from the studies were categorized and analyzed based on variable type. Categorical variables (e.g., sentinel lymph node detection rate, sensitivity, false-negative rate) were reported as proportions or percentages. Continuous variables (e.g., number of lymph nodes identified) were presented using means or medians, depending on the data provided by each study. When appropriate, ranges were indicated to reflect inter-study variability. All analyses were descriptive, and no inferential statistics were applied due to the exploratory nature of this systematic review and the limited sample sizes across studies.

### Ethical considerations

As this study involved a systematic review of published literature, no ethical approval was required.

## Results

### Study identification and characteristics

The process of article selection in this review, following PRISMA 2020 guidelines [21] is detailed in Table 1. A total of 405 records were identified through database searching. After removing 68 duplicates, 337 unique records remained. Of these, 292 studies were excluded based on title and abstract screening due to irrelevance to the study objectives, such as reviews, animal studies, or unrelated imaging techniques. The full texts of 45 articles were assessed for eligibility. Among them, 33 were excluded for the following reasons: 15 were review articles or commentaries, 9 were case series with fewer than three patients, 5 used ICG but not for sentinel lymph node mapping, and 4 involved other cancer types or procedures. Ultimately, 12 studies met al.l eligibility criteria and were included in the final qualitative synthesis. These twelve studies were ultimately included in this review and comprehensive patient characteristics, tumour stage information, dye type and dosage, tumour stages can be found in Table 3.

**Table 3:**
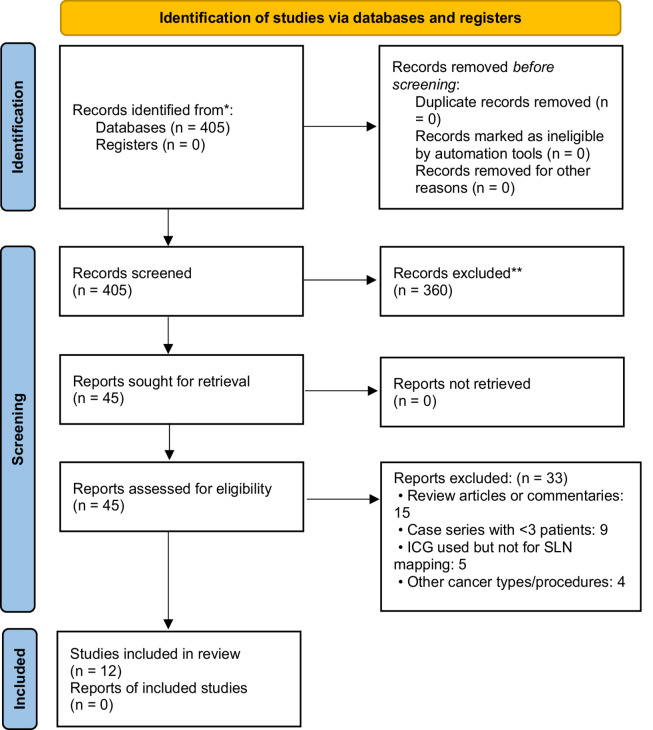
PRISMA flowchart

### Findings & outcomes

These studies, conducted between 2006 and 2024 across various countries including Japan, the UK, Germany, the Netherlands, Italy, and Romania, consistently highlight the high sensitivity and reliability of ICG in detecting SLNs.

Nagata et al. [22] in Japan studied 48 patients with colorectal tumors, achieving a sensitivity of 87.5% (42/48) using intra-operative subserosal injection with ICG Diagnogreen^®^ at 2.5 mg/mL. Kusano et al. [23]also in Japan, evaluated 26 patients with colorectal tumors and reported a sensitivity of 88.5% (23/26) using a similar protocol and ICG product.

In the UK, Cahill et al. [24] involved 18 patients and achieved a 100% sensitivity (18/18) with an endoscopic submucosal injection using ICG Pulsion^®^ at 5 mg/mL. Hirche et al. [19] in Germany studied 26 patients with colon tumors, reporting a 96.2% sensitivity (25/26) using intra-operative subserosal injection with ICG Pulsion^®^ at 5 mg/mL. Van der Pas et al. [25] in the Netherlands evaluated 14 patients with colon tumors, achieving a 100% sensitivity (14/14) with intra-operative subserosal injection of ICG Pulsion^®^ at 5 mg/mL.

Handgraad et al. [26] in the Netherlands studied 4 patients, reporting a sensitivity of 75% (3/4) using endoscopic submucosal injection with ICG Pulsion^®^ at 5 mg/mL. Chand et al. [27] in the UK evaluated 10 patients with a sensitivity of 80% (8/10) using intra-operative subserosal injection with ICG Pulsion^®^ at 5 mg/mL. Currie et al. [18]also in the UK, achieved a 90% sensitivity (27/30) in 30 patients using endoscopic submucosal injection with ICG Pulsion^®^ at 5 mg/mL.

Ankersmit et al. [28] in the Netherlands combined intra-operative subserosal and endoscopic submucosal injections, achieving an 89.7% sensitivity (26/29) in 29 patients using ICG at 25 mg/mL diluted in human albumin. Carrara et al. [29] in Italy evaluated 92 patients with intra-operative subserosal injection of ICG at 5 mg/mL, reporting a high sensitivity of 96.7% (89/92).

Staniloaie et al. [30] in Romania reported a lower sensitivity of 56.5% (13/23) in 23 patients using intra-operative subserosal injection with ICG at 2.5 mg/mL. Lastly, Sikkenk et al. [7] in the Netherlands studied 10 patients, achieving a 100% sensitivity (10/10) using endoscopic submucosal injection with ICG Verdye^®^.

The included studies employed various ICG injection techniques, including submucosal, subserosal, and peritumoral approaches. Timing of administration ranged from intraoperative to 24 h preoperatively. Fluorescence visualization was performed using laparoscopic systems in 9 studies and robotic systems in 3 studies (Table 4). Details of injection timing and visualization platforms are summarized in Table 4. Overall, these studies underscore the high sensitivity of ICG in SLN mapping, with a majority of studies using intra-operative subserosal injections and a few employing endoscopic submucosal injections. The studies consistently demonstrate the efficacy of ICG, supporting its use as a reliable tool for enhancing the accuracy of cancer staging and improving surgical outcomes. The collective findings from these twelve studies provide relative robust evidence for the effectiveness of ICG in SLN mapping across different protocols and patient populations.

**Table 4:** Summary of injection protocols, ICG dosage, timing of injection, and visualization platforms across the included studies

Study (author)	Year	Country	Study Design	Definition of SLN	Localisation of the tumor	Stage TNM I-II	Stage TNM III-IV	Patients (*n*)	Dye product (if known) & posology (mg.mL-1)	Injection protocol	TIMING OF ICG INJECTION	VISUALIZATION PLATFORM	Sensitivity
Nagata et al. [22]	2006	Japan	Prospective - Monocentric cohort study	Yes	Colorectal	37	11	48	ICG Diagnogreen^®^ (Dai-Ichi Pharmaceutical Co., Ltd., Tokyo, Japan) & 5 mg/ml	Intra-operative subserosal	Intra-operative	Laparoscopic NIR	42/48
Kusano et al. [23]	2008	Japan	Prospective - Monocentric cohort study	Yes	Colorectal	NR	NR	26	ICG Diagnogreen^®^ (Dai-Ichi Pharmaceutical Co., Ltd., Tokyo, Japan) & 5 mg/ml	Intra-operative subserosal	Intra-operative	Laparoscopic NIR	23/26
Cahill et al. [24]	2011	UK	Prospective - Monocentric cohort study	Yes	Colorectal	NR	NR	18	ICG Pulsion^®^ (Pulsion Medical Systems, Munich, Germany) & 2.5 mg/ml	Endoscopic submucosal	0–24 h pre-op	Firefly (robotic)	18/18
Hirche et al. [19]	2012	Germany	Prospective - Monocentric cohort study	Yes	Colon	NR	NR	26	ICG Pulsion^®^ (Pulsion Medical Systems, Munich, Germany) & 5 mg/ml	Intra-operative subserosal	Intra-operative	Laparoscopic NIR	25/26
van der Pas et al. [25]	2013	Netherlands	Prospective - Monocentric cohort study	Yes	Colon	4	10	14	ICG Pulsion^®^ (Pulsion Medical Systems, Munich, Germany) & 2.5 mg/ml	Intra-operative subserosal	Intra-operative	Laparoscopic NIR	14/14
Handgraad et al. [26]	2015	Netherlands	Prospective - Monocentric cohort study	Yes	Rectum	2	3	5	ICG Pulsion^®^ (Pulsion Medical Systems, Munich, Germany) & 5 mg/ml	Endoscopic submucosal	Pre-operative	Laparoscopic NIR	3/4
Chand et al. [27]	2017	UK	Prospective - Monocentric cohort study	Yes	Colon	3	7	10	ICG Pulsion^®^ (Pulsion Medical Systems, Munich, Germany) & 0.5 mg/ml/1 mg/ml/1.6/ml	Intra-operative subserosal	Intra-operative	Laparoscopic NIR	8/10
Currie et al. [18]	2017	UK	Prospective - Monocentric cohort study	Yes	Colon	30	0	30	ICG Pulsion^®^ (Pulsion Medical Systems, Munich, Germany) & 5 mg/ml	Endoscopic submucosal	Pre-operative	Firefly (robotic)	27/30
Ankersmit et al. [28]	2019	Netherlands	Prospective - Monocentric cohort study	Yes	Colon	14	15	29	ICG 25 mg ICG diluted in 1.0 ml human albumin (20%) and 9.0 ml NaCl (0.9%).	Intra-operative subserosal & endoscopic submucosal	Intra-operative	Laparoscopic NIR	26/29
Carrara et al. [29]	2020	Italy	Prospective - Monocentric cohort study	Yes	Colorectal	46	46	92	ICG & 5 mg/ml	Intra-operative subserosal	Intra-operative	Laparoscopic NIR	89/92
Staniloaie et al. [30]	2022	Romania	Prospective - Monocentric cohort study	Yes	Colorectal	3	20	23	ICG & 2.5 mg/ml	Intra-operative subserosal	Intra-operative	Laparoscopic NIR	13/23
Sikkenk et al. [7]	2023	Netherlands	Prospective - Monocentric cohort study	Yes	Colon	10	0	10	Indocyanine Green (ICG) (Verdye, Diagnostic Green GmbH, Aschheim-Dornach, Germany) & 5 mg/ml	Endoscopic submucosal	Pre-operative	Firefly (robotic)	10/10

## Discussion

Although promising, the use of fluorescent indicators for SLN mapping in colorectal cancer remains experimental. Current studies, mostly small and heterogeneous, limit the ability to draw firm conclusions regarding its diagnostic accuracy or clinical utility. Additionally, when implemented for smaller colon tumors categorized as T1-T2, the approach demonstrates enhanced accuracy and sensitivity rates. Further investigations into the application of Indocyanine Green (ICG) through submucosal injections suggest the potential for increased precision and sensitivity in detecting SLN.

### “Standardizing” the type of injection of the ICG dye

In the pursuit of optimizing Sentinel Lymph Node (SLN) mapping in colorectal surgery through the application of Indocyanine Green (ICG), the nuanced differences in injection techniques such as subserosal, submucosal, and peritumoral, along with their respective dosages, emerge as pivotal factors in refining the procedure’s accuracy and reproducibility [31, 32]. The subserosal technique, often employed with a dosage range of 0.5 to 2.5 mg of ICG, involves injecting the dye directly beneath the serosa close to the tumor, aiming to delineate the lymphatic pathways leading to the sentinel nodes. This method is particularly favored for its direct approach, potentially enhancing the visibility of lymphatic drainage patterns.

The submucosal technique, which may utilize a similar dosage, administered either endoscopically prior to surgery or directly during the operation, targets the submucosal layer surrounding the tumor. This approach is thought to provide a more immediate entry of ICG into the lymphatic system, thereby facilitating a clearer and more direct mapping to the sentinel nodes. It’s valued for its ability to closely mimic the natural lymphatic drainage from the tumor, offering a potentially higher detection rate of SLN. Some authors suggest the feasibility of an endoscopy prior to an oncological surgery is not always easy and is also associated with morbidities [5, 6, 33, 34]. However, the technique presents several challenges, especially in nowadays minimally invasive settings. Precise injection can be technically demanding, requiring advanced laparoscopic or robotic skills to avoid unintentional dye spillage, which may obscure the visualization of lymphatic channels or contaminate the surgical field. Additionally, inadvertent injection into blood vessels or excessive manipulation of the tumor can increase the risk of tumor cell dissemination.

The peritumoral injection method, where ICG is injected around the tumor site, utilizes dosages that can vary slightly but generally falls within the range used for subserosal and submucosal injections. This technique is designed to encircle the tumor with the fluorescent marker, aiding in a comprehensive visualization of the lymphatic outflow and enhancing the identification of sentinel nodes from multiple angles. It’s particularly useful in cases where the tumor’s lymphatic drainage pattern is complex or not well-defined in “abnormal” or “aberrant” drainage territories.

The choice of technique and dosage not only depends on the tumor’s location and the surgeon’s preference but also on the objective to achieve the most effective lymphatic mapping while minimizing the risk of missing micrometastases or “skipped metastasis” [8, 32, 35–37]. An additional potential benefit of SLN mapping using ICG fluorescence is the detection of these skipped metastases. These represent lymph node metastases that bypass expected proximal nodes, potentially leading to understaging and incomplete lymphadenectomy in conventional resections. Since ICG follows the functional lymphatic drainage in real-time, it may help identify such aberrant pathways and metastatic nodes outside standard resection fields, thus improving staging accuracy. However, the current data are insufficient to quantify the actual impact of ICG on the detection of skip metastases in colorectal cancer.

Despite the potential benefits of each method, the lack of standardization across the board results in variability in the procedure’s efficacy, underscored by differing rates of SLN detection and false negatives. By establishing a consensus on the optimal approach, including the injection technique and precise ICG dosages tailored to specific tumor characteristics and locations within the colorectal region, the surgical community can enhance the SLN mapping’s accuracy. Such standardization would ensure that the method’s application in clinical practice is both reliable and effective, thereby improving staging accuracy and the subsequent tailoring of treatment strategies for colorectal cancer patients. A particular challenge in SLN mapping is the identification of aberrant lymphatic drainage pathways, which may lead to sentinel nodes located outside expected anatomical and surgical regions. These nodes, if not adequately considered, could contribute to false-negative results and impact staging accuracy. Although intraoperative fluorescence imaging can help detect such atypical drainage routes, their clinical significance remains debated. Studies report variable rates of metastasis in aberrant SLNs, raising the question of their systematic inclusion in lymphatic mapping protocols. Further research is needed to determine the true impact of these nodes on oncological outcomes and to establish clear guidelines for their identification and management in clinical practice.

### Future insights & limitations

The prevailing surgical approach entails a segmental resection that includes the excision of nearby lymph nodes, a process that carries a significant risk of post-surgery complications. However, if the Sentinel Lymph Node (SLN) technique is validated as an effective method for assessing the lymph nodes in cases of colon cancer, it could pave the way for local resections through less invasive methods or endoscopic surgeries coupled with the targeted removal of the SLN as already described in upper gastric cancers [10, 38–41]. This would involve the pre-surgical injection of indocyanine green around the cancerous area. The advent of robot-assisted surgeries introduces cutting-edge tools that can be seamlessly integrated, such as the use of the Firefly camera-equipped da Vinci^®^ Robotic Surgical System for fluorescence-guided procedures. Specifically, for early-stage (T1-T2) colon cancers, a combined approach could be employed where indocyanine green is injected near the tumor endoscopically. Concurrently, the surgeon can locate the SLN from within the abdomen using the da Vinci system’s near-infrared technology Firefly^®^ [42–44]. This innovative method could revolutionize the treatment of small colon tumors, offering an even more minimally invasive alternative to traditional segmental resections. Ultimately, the move towards standardizing ICG injection methods for SLN mapping in colorectal surgery is a complex endeavor that requires ongoing research, collaboration, and consensus-building among experts. As more data become available from comparative studies examining the outcomes of different techniques and dosages, a more refined and evidence-based protocol can be developed, paving the way for improved patient outcomes through more precise and reliable lymphatic mapping.

This systematic review suggests promising outcomes; however, multiple aspects must be considered for a comprehensive evaluation. Primarily, the quantitative analysis was confined to a select few studies, raising concerns about the potential for increased bias. This is particularly evident in the risk of publication bias, as the analysis was limited to published articles, potentially overlooking unpublished research that might present varying results. The ability to differentiate between T1-T2 and T3-T4 patients was an advantage of including these specific studies. Despite attempts to incorporate a broader range of studies by reaching out to all respective authors, the exclusion of some studies was inevitable. The minimal number of studies involved, coupled with the low number of participants in each study, led to a low event rate for sensitivity. This limitation suggests that sensitivity is an unreliable metric under these circumstances, proposing that the accuracy rate could be a more robust indicator of the SLN procedure’s effectiveness in small sample sizes. Another limitation of this review is the inclusion of studies involving stage IV colorectal cancer patients. These patients have a high likelihood of lymph node involvement, which may lead to an overestimation of sentinel lymph node positivity rates and reduce the discriminatory value of SLN mapping. Conversely, in early-stage disease (stage I), the SLN positivity rate may be close to zero. This heterogeneity in disease stage may have impacted the pooled interpretation of sensitivity and accuracy. Additionally, the included studies reported sentinel lymph node mapping for tumors located in various segments of the large bowel, including the colon and rectum. The mesorectum represents a distinct embryological and anatomical structure with a specific lymphatic drainage pattern, which differs significantly from that of the colon. This heterogeneity in tumor location may influence the detection rate, lymphatic mapping accuracy, and the oncologic relevance of the SLN findings, particularly in rectal cancer, where mesorectal excision and local treatment strategies play a critical role. It is also important to consider tumor-related biological factors, such as sidedness and microsatellite instability (MSI) status. Right-sided tumors and MSI-high colorectal cancers have been associated with distinct lymphatic drainage patterns and a higher incidence of aberrant nodal spread, including skip metastases. These factors may affect both the feasibility and the prognostic value of SLN mapping. Future research should stratify outcomes by these molecular and anatomical subtypes to better understand the true clinical utility of ICG-guided SLN detection.

The need for validation of these metrics in larger, more diverse datasets becomes apparent to mitigate these issues. Furthermore, the small size of the included studies often implies a learning curve associated with the SLN procedure, which may introduce additional bias affecting the study outcomes. This learning curve can significantly skew the results, highlighting the necessity for incorporating more extensive studies to better understand the technique’s true efficacy. Additionally, the routine use of ICG for SLN mapping in colorectal cancer introduces challenges, such as prolonged surgical times and logistical complexities, particularly when coordination with gastroenterologists for preoperative endoscopy is required. These added steps, combined with a potentially limited therapeutic impact, underscore the importance of further investigation before widespread adoption of this approach.

The overall methodological quality of the included studies was limited, as they predominantly consisted of case series or retrospective analyses with small sample sizes. These study designs inherently lack the robustness of prospective, randomized trials and are more susceptible to bias, thereby reducing the reliability and generalizability of their findings. This underscores the pressing need for future research characterized by larger sample sizes, prospective methodologies, and rigorous quality standards. Although the primary objective of this review was to qualitatively assess the feasibility and current application of ICG for SLN mapping in colorectal cancer, no pooled quantitative analysis was performed due to significant heterogeneity across studies in terms of injection protocols, timing, ICG dosage, patient stages, and outcome definitions. As a result, the lack of a cumulative sensitivity or detection rate estimate limits the ability to derive generalizable performance metrics. This limitation should be addressed in future systematic reviews or prospective multicenter trials using standardized protocols. Such studies would provide more robust evidence, enabling the development of stronger conclusions and potentially influencing clinical practice guidelines. Feasibility and relevance of conducting large-scale prospective trials should be carefully weighed against the preliminary findings available. If current data suggest a strong trend in favor of a particular approach with consistent results across studies, it may be more appropriate to refine existing methodologies rather than initiate extensive new trials. Conversely, if significant uncertainties remain—such as high variability in SLN detection rates or unresolved questions about clinical benefit—then further prospective research remains essential to validate these findings and guide future practice. To definitively assess how sensitive the SLN identification process is, it would be valuable to conduct a forward-looking study that includes a broad spectrum of patients. This study should concentrate on the submucosal administration of ICG and its effectiveness specifically in identifying SLN in smaller colon tumors, categorized as T1-T2.

## Conclusion

In the current landscape of surgical oncology research, the integration of near-infrared (NIR) fluorescence imaging for sentinel lymph node mapping (SLNM) in colon cancer represents a frontier of innovation, albeit with emergent evidence. The literature underscores a critical need for the refinement of procedural protocols to enhance the reliability and application of this technique. Future investigative efforts must prioritize the delineation of early-stage neoplasms, with a targeted focus on optimizing tracer formulations, refining injection methodologies, and advancing the capabilities of real-time optical guidance systems for precise sentinel lymph node identification.

Furthermore, the application of SLNM within the context of colon cancer surgery introduces unique anatomical and procedural intricacies, distinguishing it from its utilization in other malignancies. These complexities necessitate a substantive accumulation of specialized surgical proficiency and technological adeptness. In anticipation of SLNM’s inclusion within the standard of care for colon cancer treatment, comprehensive patient-centric research is imperative. Such studies should aim to validate the clinical utility, safety, and efficacy of SLNM, ensuring its feasibility and effectiveness in enhancing oncological outcomes.

This perspective advocates for a concerted effort within the surgical community to advance the evidence base through meticulously designed large-scale clinical trials. Establishing robust procedural standards and fostering surgical expertise are paramount to unlocking the full potential of NIR fluorescence imaging in transforming the paradigms of colon cancer surgery. While ICG-based SLNM represents a promising tool, current evidence remains insufficient to support its widespread adoption. Larger, high-quality studies are essential to confirm its clinical benefits, optimize technical protocols, and clarify its potential role within established colorectal cancer treatment pathways.

## Data Availability

No datasets were generated or analysed during the current study.
